# Application of Graph Theory for Identifying Connectivity Patterns in Human Brain Networks: A Systematic Review

**DOI:** 10.3389/fnins.2019.00585

**Published:** 2019-06-06

**Authors:** Farzad V. Farahani, Waldemar Karwowski, Nichole R. Lighthall

**Affiliations:** ^1^Computational Neuroergonomics Laboratory, Department of Industrial Engineering and Management Systems, University of Central Florida, Orlando, FL, United States; ^2^Department of Psychology, University of Central Florida, Orlando, FL, United States

**Keywords:** brain connectivity, functional connectivity, effective connectivity, fMRI, brain networks, graph theory, small-world, connectome

## Abstract

**Background:** Analysis of the human connectome using functional magnetic resonance imaging (fMRI) started in the mid-1990s and attracted increasing attention in attempts to discover the neural underpinnings of human cognition and neurological disorders. In general, brain connectivity patterns from fMRI data are classified as statistical dependencies (functional connectivity) or causal interactions (effective connectivity) among various neural units. Computational methods, especially graph theory-based methods, have recently played a significant role in understanding brain connectivity architecture.

**Objectives:** Thanks to the emergence of graph theoretical analysis, the main purpose of the current paper is to systematically review how brain properties can emerge through the interactions of distinct neuronal units in various cognitive and neurological applications using fMRI. Moreover, this article provides an overview of the existing functional and effective connectivity methods used to construct the brain network, along with their advantages and pitfalls.

**Methods:** In this systematic review, the databases Science Direct, Scopus, arXiv, Google Scholar, IEEE Xplore, PsycINFO, PubMed, and SpringerLink are employed for exploring the evolution of computational methods in human brain connectivity from 1990 to the present, focusing on graph theory. The Cochrane Collaboration's tool was used to assess the risk of bias in individual studies.

**Results:** Our results show that graph theory and its implications in cognitive neuroscience have attracted the attention of researchers since 2009 (as the Human Connectome Project launched), because of their prominent capability in characterizing the behavior of complex brain systems. Although graph theoretical approach can be generally applied to either functional or effective connectivity patterns during rest or task performance, to date, most articles have focused on the resting-state functional connectivity.

**Conclusions:** This review provides an insight into how to utilize graph theoretical measures to make neurobiological inferences regarding the mechanisms underlying human cognition and behavior as well as different brain disorders.

## Introduction

The human brain comprises ~86 billion neurons connected through ~150 trillion synapses that allow neurons to transmit electrical or chemical signals to other neurons (Pakkenberg et al., 2003; Azevedo et al., 2009). Studies on modeling the human brain as a complex system have grown remarkably as neuroscientists seek to understand the comprehensive information underlying cognition, behavior, and perception (Bassett and Bullmore, 2006; Reijneveld et al., 2007; Bullmore and Sporns, 2009, 2012; He and Evans, 2010; Friston, 2011; Craddock et al., 2013; Park and Friston, 2013). Exploring the human brain from the viewpoint of connectivity patterns reveals important information regarding the structural, functional, and causal organization of the brain. Among the connectivity techniques, functional, and effective connectivity have been the focus of the computational studies in recent years (Friston, 1994, 2011; Farahani and Karwowski, 2018). Functional connectivity refers to the temporal correlations among spatially remote neurophysiological events, whereas effective connectivity refers to the causal interactions between neuronal units of the brain network (Friston, 1994). Computational methods for functional brain connectivity are generally divided into model-based and model-free (Li et al., 2009a). For the analysis of effective brain connectivity, methods such as Granger casualty, dynamic causal modeling, and Bayesian networks have been of interest to researchers (Friston, 2009; Zhang et al., 2015). Further, the human connectome (i.e., mapping the connectivity patterns of the human brain) has become an increasing topic of interest in the area of human neuroscience and can be studied using network science and graph theory (Sporns et al., 2005; Kelly et al., 2012; Van Essen et al., 2012; Sporns, 2013c).

The human brain is one of the most complex networks in the world, and studies on its static and dynamic properties have undergone explosive growth in recent years (Bullmore and Sporns, 2012; Sporns, 2013b; Kriegeskorte and Douglas, 2018). The advances in graph theory and network neuroscience (i.e., the study of the structure or function of the nervous system) offer an opportunity to understand the details of this complex phenomenon and its modeling (Vecchio et al., 2017; Sporns, 2018). Graph theoretical approaches have set up a mathematical framework to model the pairwise communications between elements of a network. In human neuroscience, graph theory is generally applied to either functional or effective connectivity. However, most studies have been devoted to functional connectivity (Bullmore and Sporns, 2009; Goldenberg and Galván, 2015).

Graph-based network analysis reveals meaningful information about the topological architecture of human brain networks, such as small-worldness, modular organization, and highly connected or centralized hubs (Bullmore and Sporns, 2009, 2012; He and Evans, 2010; Meunier et al., 2010; Bullmore and Bassett, 2011; van den Heuvel and Sporns, 2013). Small-worldness is a property of some networks in which most nodes are not neighbors of each other but can be reached from every other node by a small number of steps. This characteristic is well suited to the study of complex brain dynamics, and it confirms efficient information segregation and integration in the human brain networks with low energy and wiring costs (Watts and Strogatz, 1998). Recent studies demonstrate that the small-world property of brain networks experiences topological alterations under different cognitive loads and during development (Bassett et al., 2011; Braun et al., 2015; Cao et al., 2016; Liang et al., 2016), as well as in neurological and mental disorders (Xia and He, 2011; Fornito et al., 2012; Filippi et al., 2013; Dai and He, 2014; Stam, 2014; Fornito and Bullmore, 2015; Gong and He, 2015; Abós et al., 2017; Fleischer et al., 2017; Hojjati et al., 2017; Jalili, 2017; Miri Ashtiani et al., 2018). These alterations may provide novel insights into the biological mechanisms underlying human cognition, as well as health and disease.

Recent advances in neuroimaging have enabled mapping of the human connectome in different applications (Van Essen et al., 2012; Fornito et al., 2015). Brain function can be localized through neuroimaging techniques that assess changes in metabolism via positron emission tomography (PET) or changes in blood oxygenation level-dependent (BOLD) responses via fMRI. Structural pathways can be captured using diffusion tensor imaging (DTI), in which MRI is applied to trace white matter tracts. Finally, the timing of brain activity and its locus can be determined from electroencephalogram (EEG) or magnetoencephalogram, which respectively, measure electrical and magnetic signals outside the skull. Used separately or together, these techniques constitute the neuroimaging toolkit of scientists investigating the physiology of human brain networks (Chugani et al., 1987; Ogawa et al., 1990; Pfurtscheller and Lopes, 1999; Le Bihan et al., 2001). Among them, fMRI and PET offer a relatively low temporal resolution but have a significant spatial resolution, making them particularly useful for determining where neural signals are generated (Mehta and Parasuraman, 2013). However, PET scanning can measure the blood flow changes in an area of ~5–10 cubic millimeters while fMRI can resolve down to 3 cubic millimeters and even lower. Moreover, PET scanning is much more expensive than fMRI and requires radioactive isotopes to work (Friston et al., 1996). During the last two decades, there has been an explosion of fMRI studies mapping neural functions to distinct parts of the brain at rest or during task performance (Greicius et al., 2003), however, more attention has been directed toward resting-state fMRI (rs-fMRI) data (Lee et al., 2013).

The main purpose of this paper is to review the recent studies utilizing graph-based methods to analyze connectivity patterns in the human brain network using fMRI data. We expect to see whether the recognition of brain connectivity properties by graph theory (as measured by fMRI) has been effective in understanding the mechanisms underlying human cognition compared to the traditional approaches. The remaining sections are organized as follows. Section Methodology presents the methodology and criteria used for selecting papers to be studied in the current paper, as well as data synthesis and validity risk assessment. Section Theoretical Background: Connectivity Patterns Using fMRI first summarizes existing methods for examining the brain network connectivity, which are categorized into functional and effective patterns (3.1 and 3.2), then, focuses on the graph-theoretic concepts required for analyzing the brain connectivity architecture (3.3). Section Results provides the results of literature search, study characteristics, validity assessment of the considered studies, as well as a general overview of the selected articles. Then, section Discussion discusses the potential implications and applications of graph theory in human cognition (5.1), as well as common neurological illnesses (5.2). Finally, section Challenges and Future Directions highlights challenging issues and future perspectives in this rapidly growing field.

## Methodology

This systematic review was conducted based on the PRISMA (Preferred Reporting Items for Systematic Reviews and Meta-Analyses) guidelines (Moher et al., 2010). The starting point for this systematic review was a protocol where the research questions and the search strategy were specified to reduce the effect of research expectations on the review. Furthermore, the literature searches and systematic review adhered to the Cochrane Collaboration guidance (Higgins et al., 2011), to minimize the risk of bias and error.

### Research Questions

Based on the objectives of this systematic review described in the abstract, the following research questions were derived and form the basis of this literature review:

- RQ1: How has the computational methods for modeling the brain connectivity patterns using fMRI evolved?
- RQ2: How can research of mapping the human connectome using fMRI be classified?
- RQ3: What is the significance of graph-based approaches among the identified toolkit for brain connectivity analysis?
- RQ4: With the advent of graph theory in cognitive neuroscience, what applications have been studied in modeling human cognition and psychiatric disorders?
- RQ5: What can be learned from current graph-based research in human connectome that will lead to topics for further investigation?

### Search Strategy

The search strategy was able to first explore the search space properly, and secondly, exploit the relevant material with a rigorous evaluation process. Current and seminal research literature in the realm of fMRI brain connectivity focusing on graph-based methods including peer-reviewed journal articles, textbooks, reference books, proceedings, and conference presentations were considered key sources for this systematic review. During the exploration phase, the bibliographic search was carried out using a list of academic databases and search engines such as Science Direct, Scopus, arXiv, Google Scholar, IEEE Xplore, PsycINFO, PubMed, and SpringerLink. To meet the eligibility criteria for creating search space, articles must have been published after 1990, the time when fMRI technique was invented, with the following keyword combinations in the title, keywords or abstract: (“graph theory” or “graph analysis” or “network analysis” or “connectome” or “connectomics” or “small-world” or “modularity” or “topological change” or “topological pattern” or “functional connectivity” or “effective connectivity” or “brain connectivity” or “connectivity analysis” or “brain network” or “network connectivity” or “functional network”) and (“fMRI” or “functional MRI” or “functional magnetic resonance imaging”). These criteria resulted in a narrowing of the focus to identify the population addressing the research questions.

### Eligibility Criteria

Published original articles with the following features were included in the current study: (a) be written in English; (b) be peer reviewed; (c) identify, describe, or use empirical and/or modeled graph-based methods to quantify and/or compare connectivity patterns in the human brain network; (d) be applied to fMRI data. Other exclusion criteria were: (a) book chapters; (b) papers which upon review were not related to the research questions; (c) opinions, viewpoints, anecdotes, letters, and editorials. Two authors (FVF and WK) independently screened the titles and abstracts to find the relevant papers based on the inclusion and exclusion criteria and any discrepancies were resolved through discussion.

### Quality Assessment

Risk of bias in individual studies was assessed by two authors independently (FVF and WK) using the Cochrane Collaboration's tool (Higgins et al., 2011). The following domains were evaluated: random sequence generation, allocation concealment, blinding of participants, blinding of outcome assessment, incomplete outcome data, selective outcome reporting. To evaluate the quality of evidence across studies, we examined for lack of completeness (publication bias) and missing data from the included studies (selective reporting bias). The risk of missing studies is heavily dependent on the selected keywords and the limitations of the applied search engines. To mitigate this risk, a well-known and heavily cited set of papers was employed to construct the keyword search list in an iterative process. Accordingly, a Pareto analysis of the top keywords was conducted to assess the quality of selected keywords in search strategy.

An important concern to the validity of evidence across studies is the issue of limited attention span (i.e., the length of time a person can concentrate on a task without becoming distracted) for reviewing the sheer volume of identified scientific articles. To put it another way, the likelihood of erroneously omitting relevant articles as well as information from the included studies increases due to the repetitive and monotonous nature of reviewing a large number of papers for content under perceived and/or real-time constraints. Reduction of this risk was achieved by breaking up the articles into controllable, discrete quantities of 20–40 articles depending on article length, and providing sufficient time separation between reviews. Moreover, to prevent the formation of taxonomy with insufficient breadth when categorizing selected articles, an iterative content analysis method was employed to assure adequate classes for every new concept encountered in the literature review.

## Theoretical Background: Connectivity Patterns Using fMRI

**Figure 1:**
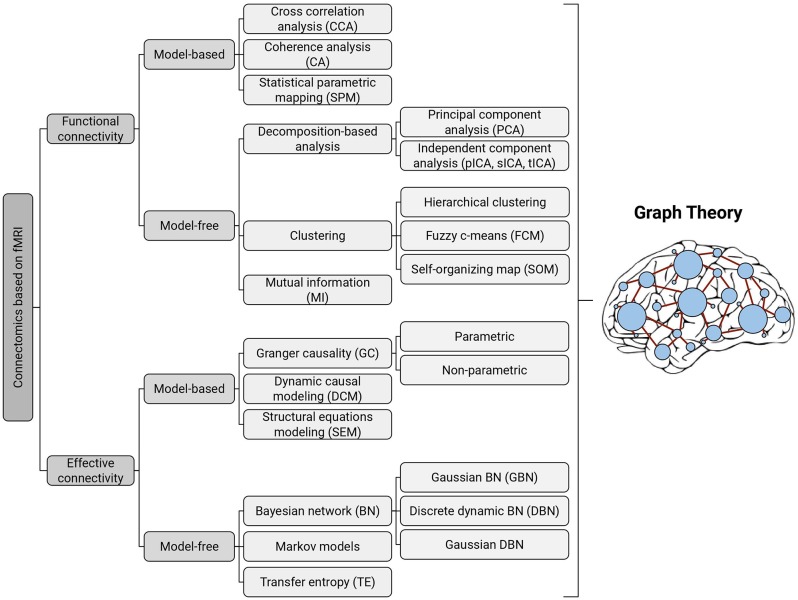
Taxonomy of existing methods for modeling functional and effective connectivity patterns using fMRI. Each of the identified methods can be represented in terms of a graph, where the nodes correspond to cortical or subcortical regions and the edges represent (directed or undirected) connections (Bullmore and Sporns, 2012); thereby all of them can be further examined with graph-theoretic measures.

Brain connectivity investigations using fMRI time-series were initiated in the mid-1990s and provided a new tool for researchers, especially neuroscientists, to study the human brain network with high precision. Computational methods available for brain connectivity are divided into two general categories: functional connectivity and effective connectivity (Friston, 1994, 2011). Briefly, functional connectivity provides information about the statistical dependencies or temporal correlations between spatially remote neurophysiological events, whereas effective connectivity is concerned with the directed influence of brain regions on each other (Friston, 2011). In the following, we will review the computational methods that are presented in the literature for investigating both types of connectivity with a greater focus on graph theoretical approaches in separate sections (Figure 1).

### Functional Connectivity

Functional connectivity refers to the temporal correlations between BOLD signals from spatially remote brain regions (Friston et al., 1993; Lee et al., 2003). Functional connectivity methods in fMRI studies are broadly divided into model-based (e.g., cross-correlation, coherence analysis, and statistical parametric mapping) and model-free (e.g., decomposition-based analysis, clustering, and mutual information) groups.

#### Model-Based Methods

Model-based methods typically identify brain connectivity networks by selecting one or more “seed” regions and then determining whether there is a linear link between seed regions and other regions using predefined criteria (Li et al., 2009a). Despite their widespread use and simple interpretation in identifying functional connectivity, the requirement for prior knowledge (particularly in rs-fMRI), dependency on the seed selection, and the inability to detect non-linear forms of interaction, restrict the discovery of all plausible functional architectures (Farahani and Karwowski, 2018).

##### Cross-correlation and coherence

Cross-correlation analysis is the most traditional method for testing functional connectivity, which is defined by measuring the correlation between the BOLD signals of any two brain regions (Cao and Worsley, 1999). The computational complexity of this method is extremely high when calculating the correlation of two series at all lags (Cecchi et al., 2007). Fortunately, a large number of fMRI studies have overcome this drawback by computing only the correlation with zero lag due to the short duration of the hemodynamic response of blood (Friston et al., 1994b; Saad et al., 2001). Moreover, correlations are sensitive to the shape of the hemodynamic response function (HRF), which causes variations across different individuals and different brain areas (Miezin et al., 2000; Lee et al., 2001). Furthermore, a high correlation may be observed among regions that practically have no blood flow fluctuations. Uncontrolled physiological noise in the brain (e.g., from cardiac and respiratory variations) can also result in high correlations between brain regions (Friston et al., 1994a). To address these problems, Sun et al. (2004) suggested a new measure, termed coherence, which is the spectral representation of correlation in the frequency domain.

##### Statistical parametric mapping (SPM)

SPM is another model-based approach used to detect region-specific effects (e.g., brain activation patterns) in neuroimaging data, such as fMRI and PET, using a combination of the general linear model (GLM) and Gaussian random field (GRF) (Friston et al., 1991). The GLM helps estimate the parameters describing the spatially continuous data by performing a univariate test statistic on each voxel. GRF theory is applied to address the multiple comparisons problem for continuous data (i.e., images) when making statistical inferences over a volume of the brain, an approach similar to the Bonferroni correction for the analysis of discrete data (Worsley et al., 1992).

#### Model-Free Methods

In contrast to seeds-based methods, model-free methods need no seeds selection. Also, model-free methods may be beneficial in studies where there are no temporal or spatial patterns, as well as in quantifying non-linear neuronal interactions (Farahani and Karwowski, 2018).

##### Decomposition-based analysis

PCA can express the fMRI data with a linear combination of orthogonal contributors that have the greatest impact on the data variance. Each contributor contains a pattern of time variability (or a principal component) multiplied by a pattern of spatial variability (or an eigen map). The created eigen maps reflect the connectivity architecture of the brain (Baumgartner et al., 2000; Worsley et al., 2005). Despite the ability to explore the whole-brain connectivity, PCA fails to detect activations when the contrast-to-noise ratio is low (Baumgartner et al., 2000). Also, how to select the optimal number of components has become an open question. Thus, PCA commonly serves as a preprocessing step in fMRI studies through dimension reduction (Li et al., 2009a). Another decomposition-based method, called independent component analysis (ICA), attracted the attention of researchers in rs-fMRI studies. The major difference between ICA and PCA is that the components in ICA should be as independent as possible (Comon, 1994; Hyvärinen and Oja, 2000). Note that a violation of component independence would reduce the efficiency of ICA (Calhoun et al., 2001). Furthermore, finding the optimal number of independent components is controversial because choosing a small number of components can have a significant effect on ICA results (Ma et al., 2007), particularly when used for decoding purposes (Douglas et al., 2011, 2013). Finally, ICA cannot discriminate between signals of interest and signals of no interest (e.g., physiological noise, unexplained signal variations), leading to overfitting and invalid assessment of statistical significance. To address this pitfall, Beckmann and Smith (2004) proposed a probabilistic ICA that allows for non-square mixing when there is Gaussian noise.

##### Clustering

The primary goal of clustering algorithms is to group voxels or regions of interest into different clusters based on the similarity between their BOLD time courses (Golay et al., 1998). Hierarchical clustering, k-means, fuzzy clustering (fuzzy c-means), self-organizing maps, graph-based, and bootstrap analysis are the most well-known algorithms used in fMRI studies (Chuang et al., 1999; Ngan and Hu, 1999; Cordes et al., 2002; Golland et al., 2008; van den Heuvel et al., 2008a; Bellec et al., 2010; Lee et al., 2012). Among these methods, the largest volume of studies utilizes hierarchical and fuzzy clustering. Hierarchical clustering seeks to construct a hierarchy of clusters based on an agglomerative or divisive strategy (Rokach and Maimon, 2005). Although this method exhibits good efficacy in the presence of respiratory or cardiac noise, its high computational complexity is a serious limitation when examining the whole brain connectivity (Cordes et al., 2002). Fuzzy c-means (FCM) is a method in which each data point has a membership value to each cluster, rather than entirely belonging to one cluster as k-means. This algorithm performs optimization by updating memberships and cluster centers until convergence (Lee et al., 2012; Lahijanian et al., 2016). It's worth noting that, given the non-Euclidean nature of MRI data, the use of Euclidean distance in FCM-based algorithms may lead to an invalid result (Farahani et al., 2015, 2018). van den Heuvel and Hulshoff Pol (2010) compared the results of clustering algorithms to those of decomposition-based methods and reported a high level of overlap. Future studies may, therefore, pay more attention to these algorithms and, by eliminating the above issues, achieve more acceptable performance in human neuroscience.

##### Mutual information (MI)

MI is an information theoretic concept that quantifies the shared information (undirected) between two random variables (Grassberger et al., 1991; Kraskov et al., 2004). Equivalently, the MI is a model-free technique that does not require any a priori assumptions about the connectivity patterns among variables, thus, it can be applied to detect both linear and non-linear correlations (Wilmer et al., 2012). Tsai et al. (1999) were among the first to present a theoretical framework for using MI to calculate the fMRI activation map. To further explore the strengths and pitfalls of this method in comparison to other functional connectivity measures, refer to Wang et al. (2014a) and Bastos and Schoffelen (2016).

### Effective Connectivity

The primary goal of effective connectivity analysis is to assess causal interactions between neuronal units of the brain network (Friston, 1994). Studies in this area help researchers better understand the mechanisms underlying neuronal dynamics (Wu et al., 2014; Farahani and Karwowski, 2018). In the following, we review the existing effective connectivity methods with their pros and cons in greater detail.

#### Model-Based Methods

Granger causality (Granger, 1969) is the most traditional model-based method for directional interactions that can be easily implemented. However, Granger causality appears to encounter difficulties when applied to fMRI data due to the underlying assumptions in its modeling (Wen et al., 2013; Dang et al., 2017). Two other model-based methods for analyzing effective connectivity are dynamic causal modeling (Friston et al., 2003) and structural equations modeling (McIntosh and Gonzalez-Lima, 1994). Despite the coherent interpretations provided by these methods, they are highly dependent on prior knowledge, so their application in analysis of rs-fMRI data is limited (Fox and Raichle, 2007).

##### Granger casualty (GC)

The core idea behind GC is that *X* “Granger-causes” *Y* if *Y* can be better predicted using the histories of both *X* and *Y* than the past of *Y* alone (Granger, 1969). Accordingly, past data from one brain region can help estimate the current state in another region. Due to the time mismatch between sampling interval and neural events, the causality method cannot be applied directly to the fMRI signals because it leads to the prediction of causal relationships in BOLD signals rather than neuronal responses (Smith et al., 2011, 2012). To tackle this issue, GC analysis is typically performed by fitting a linear vector autoregressive (VAR) to the time series (Seth, 2010; Friston et al., 2013; Seth et al., 2015). However, linear methods are not suitable for testing GC in higher moments (e.g., the variance). Non-linear and non-parametric models are used to solve this problem (Dhamala et al., 2008; Roebroeck et al., 2011). Wen et al. (2013) pointed out that several factors may hamper the neural interpretability of GC, such as low sampling rates (Lin et al., 2014), latency mismatches in HRF across distinct brain regions, and the presence of noise. Their findings reflect that GC is a viable method for analyzing fMRI signals when associated confounds are controlled.

##### Dynamic causal modeling (DCM)

DCM is based on a general bilinear state equation that quantifies how variations in neural activity in one node are affected by the activation in another node under predefined stimuli (Friston et al., 2003; Stephan et al., 2010). This equation involves a variety of information including the coupling between brain regions, changes in the coupling strength as a result of experimental conditions, and the direct effects on a region (Friston, 2009). DCM provides a powerful statistical platform that estimates the experimental modulation of both intrinsic and extrinsic connections in the brain, and the Bayesian model comparison is executed to choose the best-fitted model (Goldenberg and Galván, 2015). Perhaps the biggest disadvantage of DCM is that it is not exploratory and requires prior knowledge about the hypotheses and model specification to be implemented. However, a recent trend has emerged for comparing numerous models in a more exploratory manner using a *post hoc* analysis, wherein only the largest model is inverted while all of the reduced models would be searched quickly (Friston et al., 2011). Friston et al. pointed out that GC and DCM play complementary roles in analyzing the causal interactions (Friston et al., 2013). In fact, GC can be used generically to any specified time series to identify the coupling between neuronal units, making helpful insights into the dynamic behavior of the human brain in different situations. One might then continue effective connectivity analyses in a hypothesis-driven manner to obtain a further interpretation of the neuronal interactions using DCM (Daunizeau et al., 2011). Notably, although both build upon model selection, they have a fundamental difference. Model selection in DCM is based on a direct comparison between all models (Penny, 2012), whereas in GC this involves testing for the presence of GC followed by selecting the VAR model order using Akaike or Bayesian information criteria (Bressler and Seth, 2011).

#### Model-Free Methods

Past efforts to detect effective connectivity mostly relied on model-based methods such as GC or DCM. Model-free methods including probabilistic Bayesian networks, Markov models, and transfer entropy have been developed to determine non-linear forms of directed interactions. These methods do not require a priori assumptions on connectivity patterns due to their exploratory nature (Ramsey et al., 2010), but lagged interactions between fMRI time-courses may be a common shortcoming for most of them (Dang et al., 2017).

##### Bayesian network (BN)

BN is a probabilistic model well suited for representing the conditional dependencies over a set of random variables through a directed acyclic graph (DAG) (Friedman et al., 1997). Each edge indicates a dependency between two variables (nodes), where the lack of connection between any pair of nodes reflects conditional independence. Each node has a probability distribution: In root nodes, this is prior probability, while in child nodes this is the conditional probability (Das, 2004; Daly et al., 2011). Gaussian BN (Li et al., 2009b) and discrete dynamic BN (DBN) (Rajapakse and Zhou, 2007; Zeng and Ji, 2010) are the most commonly used techniques in this area. Due to the static nature of Gaussian BNs, they are unable to explicitly model the temporal interactions between multiple processes in different parts of the brain (Rajapakse and Zhou, 2007). Compared with Gaussian BN, discrete DBN is not limited by linear assumptions, and it can model temporal processes via a first-order Markov chain (Rajapakse and Zhou, 2007). However, the presence of multinomial distribution in the nodes of discrete DBN causes discretization of the data, leading to a huge loss of information. To overcome the primary limitations of both methods, Wu et al. (2014) proposed a method called Gaussian DBN based on a first-order linear dynamic system.

##### Transfer entropy (TE)

TE is a non-parametric approach measuring the transfer of information between joint processes based on information theory (Schreiber, 2000). Because of its non-linear nature, this method is able to properly detect directional connectivity even if there is a wide distribution of interaction delays between the two fMRI signals (Vicente et al., 2011; Sharaev et al., 2016). Although TE and GC are relatively equivalent for Gaussian variables (Barnett and Seth, 2009), TE needs much less computational time than GC for high model orders and greater numbers of nodes. In addition, TE does not assume any particular model as underlying the interactions, therefore, its sensitivity to all order correlations becomes a privilege for exploratory analyzes over GC or other model-based methods (Vicente et al., 2011; Montalto et al., 2014). However, contrary to the model-based methods, it is more difficult to interpret this measure in functional connectivity analysis due to its generality (Bastos and Schoffelen, 2016).

### Graph Theory: Analysis of the Brain as a Large, Complex Network

The first application of graph theory and network analysis can be traced back to 1736 when Leonhard Euler solved the Königsberg Bridge Problem (Euler, 1736). In this regard, a graph consists of a finite set of vertices (or nodes) that are connected by links called edges (or arcs). Following the emergence of promising results in electrical circuits and chemical structures in its early applications, graph theory has now become influential in addressing a large number of practical problems in other disciplines, such as transportation systems, social networks, big data environments, the internet of things, electrical power infrastructures, and biological neural networks (Watts and Strogatz, 1998; Boccaletti et al., 2006; Schweitzer et al., 2009).

**Figure 2:**
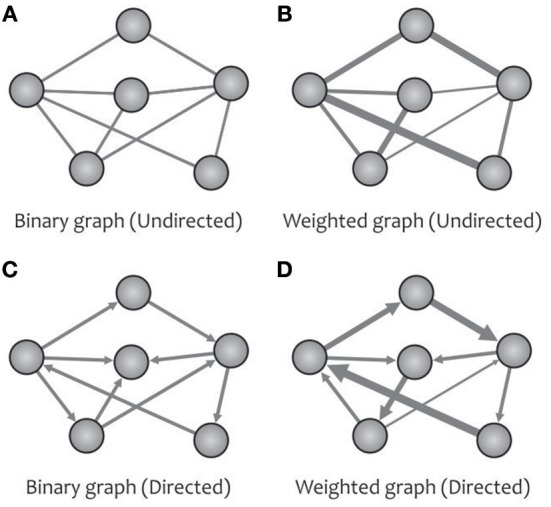
A network can be designed as binary **(A)** or weighted **(B)** graphs, and can represent the direction of causal effects **(C,D)** among different regions.

The turning point of the complex brain network studies using graph theory goes back to the introduction of the “Human Connectome” (Sporns et al., 2005). In graph theory, an N × N adjacency matrix (also called a connection matrix) with the elements of zero or non-zero indicates the absence or presence of a relationship between the vertices of a network with N nodes. By extracting different metrics from this matrix, one can obtain a topological analysis of the desired graph (e.g., the human brain network). A brain graph may be classified as either directed or undirected (Figure 2) based on whether the links between vertices carry directional information (e.g., causal interaction). Up to now, most human brain investigations have been devoted to the undirected networks because of the technical constraints surrounding the inference of directional networks (Liao et al., 2017). A brain graph can also be categorized as either weighted or binary (Figure 2) based on whether the links between vertices can take different values. For instance, in a white matter anatomical network taken by diffusion MRI, we can obtain a weighted network using various information, such as fiber number, fiber length, and fractional anisotropy (Fornito et al., 2013; Zhong et al., 2015).

In 1998, Watts and Strogatz showed that many social, biological, and geoscience-based networks have a very striking organization, called “small-world” architecture, that makes them act as regular networks, while they occasionally experience random activity (Watts and Strogatz, 1998; Figure 4C). Small-world networks represent the shortest path between each pair of nodes in the network using the minimum number of edges. In small-world networks, the clustering coefficient (also referred to as transitivity) is high, and the average path length is short. These two characteristics are the result of a natural process to satisfy the balance between minimizing the resource cost and maximizing the flow of information among the network components (Bassett and Bullmore, 2006; Meunier et al., 2010; Bullmore and Sporns, 2012; Chen et al., 2013; Samu et al., 2014). Liao et al. (2017) explained in detail why the human brain network is expected to have a small-world architecture. The metabolic and wiring costs in connections among anatomically adjacent brain areas are lower than those among distant brain regions (Bullmore and Sporns, 2012). Theoretical examinations have pointed out that the brain regions are more likely to interact with their neighboring areas to reduce the whole metabolic costs, while at the same time they need to have a small number of long-distance connections among themselves to accelerate data transmission (Sik et al., 1995; Karbowski, 2001; Bullmore and Sporns, 2012; Vertes et al., 2012; Chen et al., 2013). In agreement with theoretical studies, empirical investigations have also proved the dispersion of a few long connections among a plethora of short connections in the human brain network (Salvador et al., 2005; Hagmann et al., 2007; He et al., 2007).

The main capability of graph theory in neuroscience studies is usually unveiled after the construction of a functional brain network. Several measures can be used to assess the topological patterns of different networks such as clustering coefficient, modularity, average path, small-worldness, assortativity, and node centrality, which have been described in detail (Sporns et al., 2004; van den Heuvel et al., 2008b). Typically, one cannot claim which measures are more suitable for studying the brain network (Bullmore and Sporns, 2009), but given the complex structure of the human brain, measures that can represent the small-world properties of the brain network are of great importance (He and Evans, 2010; Liao et al., 2017). This critical property arises with the help of hubs (i.e., highly connected nodes in a network), causing the creation of local clusters (Bullmore and Sporns, 2009; Jain, 2011). In the following, we discuss how to build a brain connectivity network using fMRI data and then explain the main measures that can be extracted from the brain network with the help of graph theory.

#### Construction of Functional Brain Network Using fMRI

**Figure 3:**
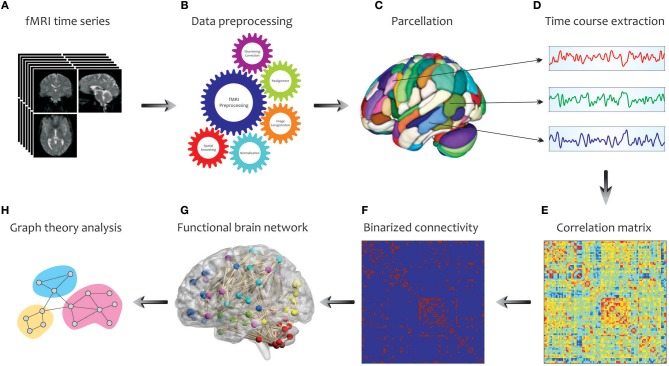
Schematic representation of brain network construction and graph theoretical analysis using fMRI data. After processing **(B)** the raw fMRI data **(A)** and division of the brain into different parcels **(C)**, several time courses are extracted from each region **(D)** so that they can create the correlation matrix **(E)**. To reduce the complexity and enhance the visual understanding, the binary correlation matrix **(F)**, and the corresponding functional brain network **(G)** are constructed, respectively. Eventually, by quantifying a set of topological measures, graph analysis is performed on the brain's connectivity network **(H)**.

In Figure 3, we illustrate the main steps used to extract a complex network from fMRI in graph theoretical analysis. Initially, a number of pre-processing steps including slice timing correction, realignment, image co-registration, normalization based on segmentation, and spatial smoothing, are performed on the acquired fMRI data. Note that, the choice and ordering of the preprocessing steps may affect the extent of final graph measures (Gargouri et al., 2018). Then, to explore the large-scale brain network, an appropriate parcellation scheme such as anatomical automatic labeling atlas is applied to divide the entire brain into several cortical and subcortical anatomical units (Tzourio-Mazoyer et al., 2002). This is followed by extracting the time series of each parcel by averaging the time courses of all voxels within that certain region. Next, one of the connectivity methods reviewed in the previous parts, such as cross-correlation, is conducted to determine the pairwise associations between the time series of brain parcels, representing the functional connectivity network (i.e., correlation matrix). A binary connectivity matrix (i.e., adjacency matrix) is then obtained by thresholding the values of the correlation matrix. Finally, key topological properties that characterize the local and global architecture of the brain network connectivity can be obtained using the Brain Connectivity Toolbox (http://www.brain-connectivity-toolbox.net/; Rubinov and Sporns, 2010). These characteristics are explained in the following.

#### Computation of Graph Measures

**Figure 4:**
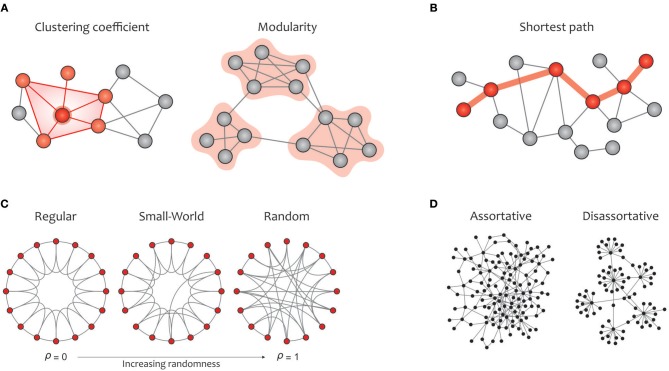
Summary of global graph measures. **(A)** Segregation measures include clustering coefficient, which quantify how much neighbors of a given node are interconnected and measures the local cliquishness (i.e., the extent to which the neighbors of a node can build a complete graph); modularity, which is related to clusters of nodes, called modules, that have dense interconnectivity within clusters but sparse connections between nodes in different clusters. On the one hand, dense communications within a certain module increase the local clustering and, consequently, enhance the efficiency of information transmission in the given module. On the other hand, a few connections between different modules integrate the global information flow, which is associated with a reduction in the average path length in the graph **(B)** Integration measure include characteristic path length, which measures the potential for information transmission, determined as the average shortest path length across all pairs of nodes. **(C)** A regular network (left) displays a high clustering coefficient and a long average path length, while a random network (right) displays a low clustering coefficient and a short average path length. A small-world network (middle) illustrates an intermediate balance between regular and random networks (i.e., they consist of many short-range links alongside a few long-range links), reflecting a high clustering coefficient and a short path length. **(D)** The assortativity index measures the extent to which a network can resist failures in its main components (i.e., its vertices and edges). Notably, communication between hubs in assortative networks leads to covering each other's activities when a particular hub crashes, but the performance in disassortative networks will drop sharply due to the presence of vulnerable hubs.

**Figure 5:**
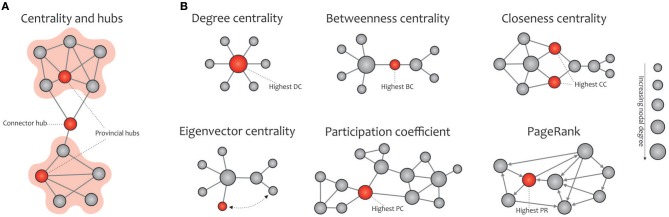
Basic concept of network centralities. **(A)** Hubs (connector or provincial) refer to nodes with a high nodal centrality, which can be identified using different measures. **(B)** The degree centrality is defined as the number of node's neighbors. The betweenness centrality measures the node's role in acting as a bridge between separate clusters by computing the ratio of all shortest paths in the network that contain a given node. The closeness centrality quantifies how fast a given node in a connected graph can access all other nodes, hence the more central a node is, the closer it is to all other nodes. The eigenvector centrality is a self-referential measure of centrality that considers the quality of a link, so that being connected to a central node increases one's centrality in turn; the red colored node is more central than the gray colored node, although their degrees are equal. The participation coefficient of a node represents the distribution of its connections among separate modules. PageRank is a variant of eigenvector centrality, used by Google Search to determine a page's importance; the PageRank of an undirected graph is statistically similar to the degree centrality, but they are generally distinct. Note that the size of the nodes in all cases is proportional to the node degree, and the red nodes (except in the eigenvalue centrality) are the most central with respect to the corresponding definition of centrality, even though their degree are low.

In this subsection, the most commonly used graph metrics for characterizing the functional brain network are described in two main groups: global and local properties. Most of these criteria are applicable to any type of binary, weighted, and directed networks. In addition to visualizing these properties in Figure 4 (global metrics) and Figure 5 (local metrics), respectively, their corresponding formulas can be accessed on https://sites.google.com/site/bctnet/measures.

##### Global properties

Global measures are primarily aimed at revealing: (a) functional segregation and (b) functional integration of information flows within the brain network; (c) small-worldness; (d) network resilience against failure (Rubinov and Sporns, 2010; Sporns, 2013a). Segregation refers to the degree to which network elements form specialized communities, and integration provides insight into the efficiency of global information communication or the ability to combine distributed information (Watts and Strogatz, 1998). Clustering coefficients and modularity are the most common metrics that quantify the properties of topological segregation in brain networks (Newman, 2004; Boccaletti et al., 2006; Rubinov and Sporns, 2010; Figure 4A). In brain networks, anatomically adjacent or functionally connected areas are generally considered as modules. Various studies have demonstrated that networks based on modular structure generally reflect the properties of small-world networks (Bullmore and Sporns, 2009; Fortunato, 2010; He and Evans, 2010; Meunier et al., 2010; Sporns and Betzel, 2016). On the other side, functional integration is typically measured by the characteristic path length that quantifies the ability for global information integration (Boccaletti et al., 2006; Rubinov and Sporns, 2010; Figure 4B). The small-world property displays an optimal balance between network segregation and integration, and is dedicated to graphs in which most nodes are not neighbors but can be reached by any other node with the minimum possible path length (Achard, 2006; Humphries et al., 2006; Humphries and Gurney, 2008; Figure 4C). Eventually, assortativity quantifies network resilience against random or deliberate damages in the main components, which is one of the most significant issues in network science (Noldus and Van Mieghem, 2014; Figure 4D).

##### Local properties

In network science, hubs refer to nodes with a high nodal centrality and thus profoundly affect the network topology. Hub nodes of a network are divided into two categories, the connector or provincial, based on the high or low participation coefficient defined for them, respectively. Connector hubs tend to interconnect nodes between different modules, while the provincial hubs are responsible for linking nodes in the same module (He et al., 2009; Power et al., 2013; Figure 5A). The easiest way to detect hubs in a network is to calculate the nodal degree, i.e., counting the edges connected to each node. Also, plotting the degree distribution *P*(*k*) of a certain network provides valuable information about the presence of hubs in it, e.g., the existence of several high degree nodes in scale-free networks is accompanied by power-law distribution (Barabási and Albert, 1999). Furthermore, other commonly used indexes for measuring the nodal centrality include betweenness, closeness, and eigenvector, participation coefficient, and PageRank (Boccaletti et al., 2006; Rubinov and Sporns, 2010; Zuo et al., 2012; Figure 5B).

## Results

### Literature Search

**Figure 6:**
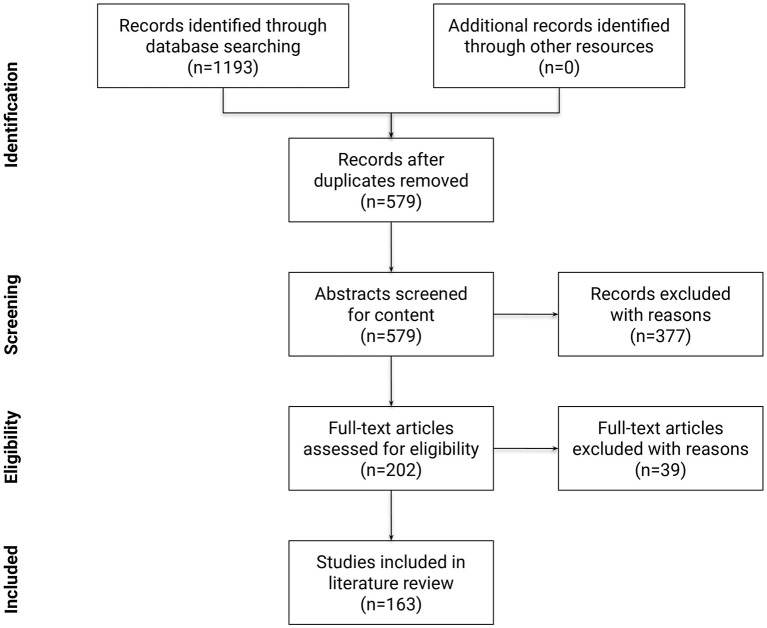
Flow diagram of the methodology and selection processes used in this review. It follows the guidelines of PRISMA (Moher et al., 2010).

Following the PRISMA guidelines (Moher et al., 2010), a summary of the identification, screening, and selection of studies for inclusion in this review is displayed in Figure 6. At the first step, 1,193 papers were identified. Next, 579 papers remained after removing duplicates. Papers published before 2005 accounted for only 5% of all papers, reflecting the novelty of the terminology and the research area. In the third step, relevant scientific articles were selected from the remaining 579 papers using a formal abstract screening process that incorporated pre-determined inclusion and exclusion criteria. Inclusion criteria at this step required the research to: (a) be written in English; (b) be peer reviewed; (c) identify, describe, or use empirical and/or modeled graph-based methods to quantify and/or compare connectivity patterns in the human brain network; (d) be applied to fMRI data. Other exclusion criteria were: (a) book chapters; (b) papers which upon review were not related to the research questions; (c) opinions, viewpoints, anecdotes, letters, and editorials. Application of inclusion and exclusion criteria at this step yielded 202 eligible articles (roughly 35% of the original papers). At the fourth step, the full text of these 202 articles were studied in detail to confirm that they met same criteria as the third step. After the fourth step, 163 publications remained for review.

### Study Characteristics

**Figure 7:**
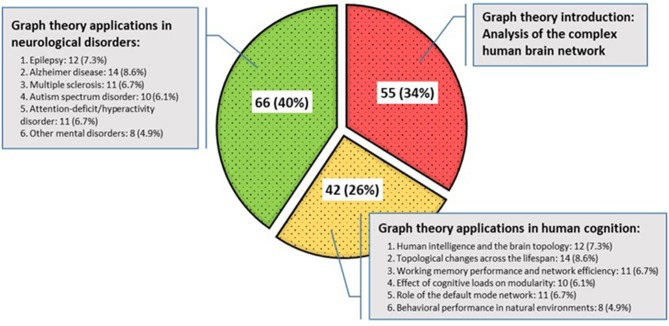
Categorization of included studies.

Sample size across studies ranged from 5 to 763 participants. The mean, mode, median, and standard deviation for the participants in all the study samples were 116.73, 40, 60, and 158.87, respectively. The included studies were published from 1998 to 2018 and organized into three taxonomies (Figure 7). The first group deals with the topological concepts of graph theory for the discovery of the brain as a large and complex network, which account for 34% of the selected articles. Then, papers that have applied graph theory in terms of human cognition and behavior for quantifying or comparing connectivity patterns in the brain network have been considered, accounting for 26% of the selected articles. Finally, applications of graph theory in mental disorders were reported, which account for 40% of the selected papers. In particular, the detailed frequency and percentage of the referenced papers in the last two categories are shown, separately.

### Quality Assessment

**Figure 8:**
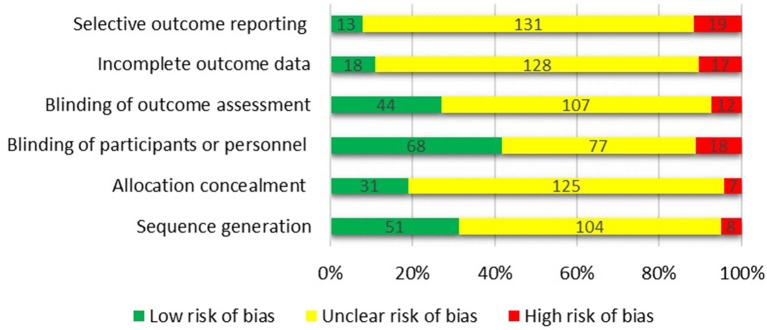
Assessing the risk of bias using the Cochrane collaboration's tool.

The Cochrane collaboration's tool (Higgins et al., 2011) was used to assess the risk of bias in each trial (Figure 8). The articles were categorized as: (a) low risk of bias, (b) high risk of bias, or (c) unclear risk of bias for each domain. Using Cochrane collaboration we judged most domains to be unclear or not reported. Eventually, the overall quality of the studies was categorized into weak, fair, or good, if <3, 3, or ≥4 domains were rated as low risk, respectively. Among 163 studies included in the systematic review, 52 were categorized as good quality, 39 were fair quality, and 72 were low quality.

### General Overview

In this part, a general overview of the selected papers is presented in terms of publication trend, keyword analysis, and frequency of authors. Such findings provide a novel perspective on the evolution of computational methods for modeling the brain connectivity patterns and the importance of graph theory among them, addressing research questions 1, 2, and 3.

**Figure 9:**
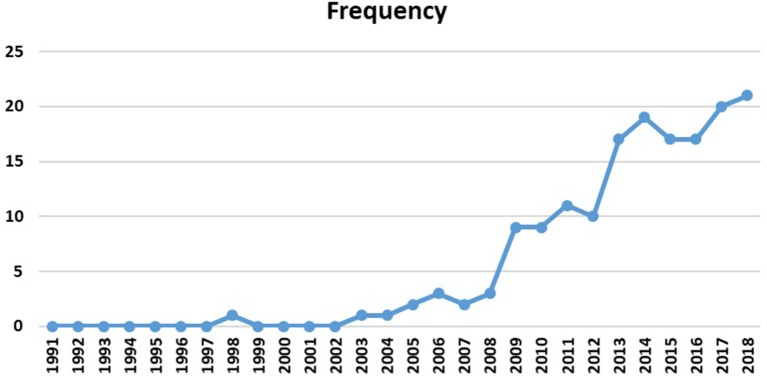
Selected papers per year (publishing trend).

To observe the evolution of the theme, Figure 9 displays the number of reviewed publications, year by year. This figure illustrates the researchers' special attention to human connectome studies, especially the emerging role of graph analysis in topological explorations of the complex brain connections since 2009. Most articles are concentrated between 2009 and 2018 (92% of the selected publications), which is expected to increase dramatically in the next years. Interestingly, the Human Connectome Project (HCP) was launched in 2009 with the National Institutes of Health sponsorship, which is in line with these findings (Nih.gov., 2009).

**Figure 10:**
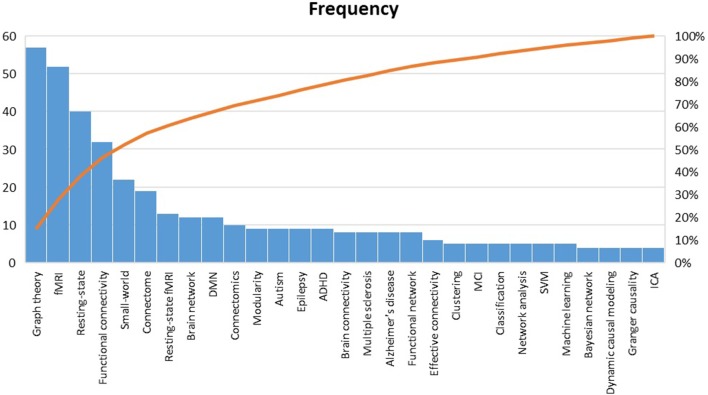
Pareto analysis of top keywords. *fMRI*, Functional magnetic resonance imaging; *DMN*, default mode network; *ADHD*, Attention-deficit/hyperactivity disorder; *MCI*, Mild cognitive impairment; *SVM*, Support vector machine; *ICA* independent component analysis.

Pareto analysis of the top keywords is shown in Figure 10. Obviously, the words “graph theory,” “fMRI,” “resting-state,” “functional connectivity,” and “small-world” were among the most used keywords in the reviewed papers (50% of the listed keywords). By this finding, it can be interpreted that those fMRI studies that have benefited from graph theory have: (a) been mostly carried out during resting-state than experimental task, which is in line with the HPC claim (Smith et al., 2013); (b) concentrated more on functional connectivity than effective connectivity; (c) considered a pivotal role for the small-world phenomenon in constructing the human brain architecture.

**Figure 11:**
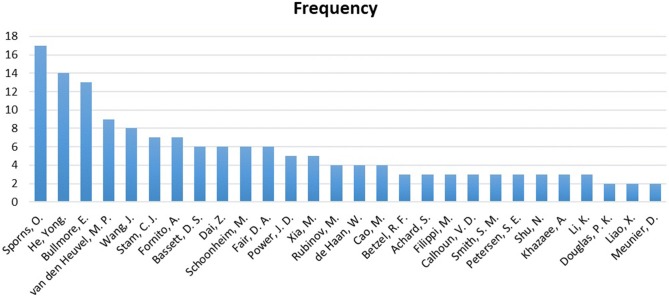
Frequency of the authors in the references.

Figure 11 displays a reference analysis through the sample. The most cited authors by the articles in our sample were Olaf Sporns, Karl Friston, Yong He, and Edward T Bullmore, with 17, 15, 14, and 13 references, respectively. Unsurprisingly, Sporns and Bullmore stand out as two of the pioneers of the network neuroscience and connectomics. It was through the study of Bullmore and Sporns (2009), entitled “Complex brain networks: graph theoretical analysis of structural and functional systems,” that complex analysis of human brain connectivity became widespread in the world.

## Discussion

Deeper discussions about the leading applications of graph theory in cognitive and behavioral topics, as well as different neurological and psychiatric illnesses are provided in two separate subsections. Considering the weaknesses and strengths of these implications provides an insight into how to utilize graph measures to make neurobiological inferences regarding the mechanisms underlying neuronal dynamics, in line with questions 4 and 5 of the research.

### Cognitive and Behavioral Applications of Graph Theory

Recent advances in neuroimaging modalities combined with graph theoretical approaches have opened new avenues toward studying the neural mechanisms underlying human cognition and behavior from the view of interregional brain interactions (Park and Friston, 2013; Pessoa, 2014; Sporns, 2014; Medaglia et al., 2015; Petersen and Sporns, 2015; Kriegeskorte and Douglas, 2018). Cognition involves a range of neuronal actions for knowledge assimilation and integration through thinking, experience, and the senses. Cognition contains manifestations of attention, comprehension, memory, decision making, reasoning, judgment, and executive functions (Mesulam, 1998). In the following, some of the applications of graph theory are presented in revealing human behavioral and cognitive performance, as well as the role of different large-scale brain networks in various conditions.

#### Human Intelligence and Brain Topology

Human intelligence refers to the marvelous and subtle function of human cognition, which is generally characterized by complex reasoning, conceptual thinking, and learning swiftly from experiences (Guilford, 1967). An early review of brain imaging studies has linked human intelligence to the structure and function of spatially distributed regions (Jung and Haier, 2007), indicating the possible importance of interactions between several regions, particularly in the frontal and parietal areas. Recently, many studies have focused on the relationship between general intellectual ability and small-world characteristics in intrinsic functional networks for describing individual differences in general intelligence (van den Heuvel et al., 2009; Langer et al., 2012; Hilger et al., 2017a). According to these studies, better intellectual performance was associated with shorter characteristic path length, the nodal centrality of hub regions in the salience network, as well as the efficiency of functional integration between the frontal and parietal areas (Jung and Haier, 2007). Through an analysis of rs-fMRI data, Wu et al. (2013) illustrated that intelligence quotient is positively correlated with nodal properties in the attention-related network and is negatively correlated with nodal properties in the default mode, emotion, and language systems. However, although these findings suggest that general intelligence is profoundly affected by the functional integration of spatially distributed regions, they could not provide sufficient information as to whether and how human intellectual performance is associated with the brain's modular architecture. To address this issue, Hilger et al. (2017b) proposed that intelligence involves the nodal characteristics of functional connectivity within and between different brain modules (especially in the parietal and frontal areas), not global modularity properties or whole-brain ratios of distinct node types.

#### Topological Changes Across the Lifespan

The human brain goes through remarkable functional changes during the lifespan, from birth to adulthood. Modeling the lifetime trajectory of the functional connectome, multiple studies detected striking age-related alterations in highly connected hub areas mainly within the default mode, attentional, sensorimotor, and visual regions via rs-fMRI (Meunier et al., 2009; Fransson et al., 2011; Hwang et al., 2013; Wu et al., 2013; Betzel et al., 2014; Cao et al., 2014b; Grayson and Fair, 2017; Finotelli et al., 2018; Gozdas et al., 2018). Most of them also reported that local efficiency and the rich club coefficient (a metric that measures the extent to which well-connected nodes also connect to each other) were incremental until adulthood in healthy subjects and then dropped with aging, while global efficiency remained almost unchanged over the lifetime regardless of the early years after birth (Gao et al., 2011). Cao et al. (2014b) further identified changes in the number and strength of connections that were created to achieve an optimal balance between the wiring costs and communication efficiency over the lifespan (Bullmore and Sporns, 2012).

Moreover, inverse trajectories of change between long and short connections suggest a continuous reorganization in the functional brain network with aging, leading to significant behavioral and cognitive differences throughout an individual's life. Regarding modularity, there are somewhat mixed findings. Some have argued for little change in modularity during brain development (Fair et al., 2009) and aging (Meunier et al., 2009), while Cao et al. reported a linear downward trend (Cao et al., 2014b). In this regard, combining other functional neuroimaging techniques, as well as performing structure-function studies, will help elucidate the neural substrates underlying cognitive and behavioral differences during developmental stages (Shah et al., 2018).

#### Working Memory Performance and Network Efficiency

Working memory is a psychological construct for the temporary storage and manipulation of the information required to perform intricate cognitive tasks such as reasoning and decision-making (Diamond, 2013). Stanley et al. (2015) compared the functionality of working memory between young and older adults in an n-back experiment by quantifying the local and global measures in their brain networks. They demonstrated that lower local efficiency corresponds to the better performance of working memory in both groups. In contrast, increasing global efficiency has been correlated with high functionality in young adults but with a slight deficiency in older adults. Seeking to prove the right intraparietal sulcus as an area responsive to manipulations of working memory load, Markett et al. (2018) used rs-fMRI to show that centrality measures in this region correlate inversely with working memory capacity. In another fMRI study, Gong et al. (2016) analyzed how active learning from action video games affected the neuroplasticity of the brain by testing the integration of working memory- (central executive) and attention-related (salience) neural networks. By assessing the graph theoretical properties between advanced and amateur players, they revealed that long-term playing would enhance the functional integration within and between working memory and attention systems.

#### Effect of Cognitive Loads on the Brain Modularity

In the last decade, studies on dynamic reconfiguration of human brain topology during different cognitive tasks have attracted widespread attention. Researchers believe that such functional brain networks adapt flexibly to their cognitive demands while preserving the modular structure (Bassett et al., 2011; Fornitoa et al., 2012; Braun et al., 2015; Liang et al., 2016). In the course of dynamic reorganization, the parietal and frontal brain regions that hold several connector (inter-modular) hubs are discerned to play crucial roles by regulating their brain-wide connections (Cole et al., 2013; Braun et al., 2015). For instance, intensifying cognitive loads during a working memory task is associated with increased integration between different modules of the brain network (Kitzbichler et al., 2011; Braun et al., 2015; Liang et al., 2016). Furthermore, flexibility and the inter-modular integration of frontal areas are associated with high performance on working memory tasks (Braun et al., 2015).

Regarding mental state analysis, notable studies have shown that modularity corresponds negatively to the level of consciousness by comparing the functional brain network in individuals who experienced non-rapid eye movement sleep and those in wakefulness (Boly et al., 2012; Tagliazucchi et al., 2013). The common point of all these findings is that an increased cognitive load or consciousness level brings about greater global integration of the neural networks (i.e., reducing the modularity coefficient). However, further studies are needed to make this claim more robust.

#### Role of the Default Mode Network in Behavioral Performance

Comparing the brain topological alterations during a cognitive task and resting-state using fMRI data helps identify areas that affect human behavioral performance. Desalvo et al. (2014) used a graph-based approach to explore variations in functional brain organization during semantic decision making compared with rest in healthy participants. They observed that differences were generally associated with the language-related and DMN regions. More importantly, they found greater intra-modular communication in these regions during decision making (i.e., a decrease in distributed connectivity), whereas the inter-modular communication was stronger at rest.

Moreover, Lin et al. (2016) analyzed whether cognitive behavior correlates with the functional connectivity of the DMN in healthy subjects, both while at rest and during an attentional task. Quantifying the static and dynamic nodal properties within the DMN, they revealed the importance of the default network, especially the posterior cingulate areas, on human cognitive performance. Finally, Sadaghiani et al. (2015) investigated the relationship between ongoing alterations in baseline connectivity patterns and behavioral performance through a continuous auditory detection task. Interestingly, their results indicated a reduction in modularity (i.e., increasing integration efficiency) before misses compared with hits and task-free rest, mostly in the DMN areas and visual networks. These findings augment our understanding about the key role of the DMN in behavioral performance at rest and during a task; however, its association with other brain regions in more complex cognitive tasks, such as reasoning and executive functions, requires further studies.

#### Behavioral Performance in Natural Environments and Everyday Settings

One of the fascinating areas of cognitive neuroscience in recent years is neoroergonomics; that is to say, the behavioral analysis of the human brain performance with regard to environments, work, technology, and everyday settings (Parasuraman and Rizzo, 2008). Qian et al. (2013) studied the topological changes of the brain connectome during passive hyperthermia using rs-fMRI data. Despite maintaining economic small-worldness in both normal and hyperthermia conditions, the brain networks of heat-exposed subjects exhibited decreased clustering coefficients, as well as decreased local efficiency and small-worldness indices, suggesting a tendency toward a random network. They also conducted an attention network test (ANT). Their findings were highly relevant to global measure alterations and pre-frontal local efficiency, indicating behavioral disorders during environmental heat exposure in executive attention but not in alerting or orienting.

Furthermore, functional imaging analyses on mental fatigue have indicated that declines in performance from fatigue are associated with brain topological alterations such as a decrease in small-world properties and global efficiency, as well as functional changes in the fronto-parietal network and connected areas in the thalamus and the striatum (Petruo et al., 2018). In particular, graph-based investigations using fMRI data express that long-range connectivity is changed when the effects of fatigue appear (Sun et al., 2014, 2017). For instance, Sun et al. (2017) studied the effects of a mid-task break on enhancing local efficiency and reported no significant impact of rest breaks on task performance. In general, such studies help to understand the neural mechanisms of fatigue; thus, by adopting a suitable recovery approach, one can try to improve human performance during cognitive tasks.

### Disorganization of Brain Networks in Neurological and Psychiatric Disorders

Disconnection in a brain made up of localized but linked specialized regions results in functional impairment, associating with atypical integration of distributed brain areas. Catani and Ffytche (2005) elaborated the rises and fall of disconnection syndromes and pointed out that many neurological disorders can be explained via these syndromes, in line with the studies of pioneers in neurology and psychiatry such as Meynert, Wernicke, and Dejerine. Studies in the field of complex brain networks have demonstrated that analyzing the network properties and metrics derived from brain topology using rs-fMRI can help neurologists distinguish patient groups from control subjects in mental disorders (Bassett and Bullmore, 2009; Wang et al., 2010; Stam, 2014; Zhou et al., 2017). In the following, several studies that have used graph theory to investigate common neurological disorders, comprising epilepsy, Alzheimer's disease (AD), multiple sclerosis (MS), autism spectrum disorder (ASD), and attention-deficit/hyperactivity disorder (ADHD), are discussed. However, other mental disorders were also found in recent graph-based literature, including schizophrenia, Parkinson's disease, insomnia, major depression, obsessive compulsive disorder (OCD), borderline personality disorder (BPD), and bipolar disorder (Armstrong et al., 2016; Kambeitz et al., 2016; Manelis et al., 2016; Xu et al., 2016; Algunaid et al., 2018; Díez-cirarda et al., 2018; Li et al., 2018; Zhi et al., 2018), but their contribution is negligible and more attention is required in future research.

#### Epilepsy

Epilepsy is a chronic neurological disorder that is accompanied by aberrations in brain activity, resulting in recurring seizures and occasionally loss of consciousness (Hauser and Hesdorffer, 1990). Temporal lobe epilepsy (TLE) is the most prevalent form of epilepsy with partial seizures (Bernhardt et al., 2015). In two interesting rs-fMRI studies using network analysis, Výtvarová et al. (2017) and Dong et al. (2016) described the contribution of basal ganglia thalamocortical circuitry to the whole-brain functional connectivity in TLE. Although the detection and removal of epileptogenic lesions are necessary for the abolition of seizures, many studies have shown that seizures in TLE originate from abnormalities in the epileptogenic network rather than from lesions (Rosenow and Lüders, 2001; Cooray et al., 2015); thus, seizure recurrence is observed following ~40% of epilepsy surgeries within 5 years (Spencer, 2002). Therefore, the application of graph theory, along with clinico-radiological findings, helps to better understand the network mechanisms behind a cognitive decline in focal epilepsies, particularly TLE, and offers promising diagnostic biomarkers (Chiang and Haneef, 2014; Onias et al., 2014; Wang et al., 2014b; Pedersen et al., 2015; Ridley et al., 2015; Iyer et al., 2018).

Vlooswijk et al. examined small-world properties in patients with TLE using rs-fMRI (Vlooswijk et al., 2011). In contrast to healthy subjects, they found a disruption of both local segregation [opposed to Wang et al. (2014b)] and global integration in patients with epilepsy. They confirmed the association between the IQ score and information processing performance, whether it is specialized or serial. The correlation between average path length and intellectual capability has been indicated by other experiments as well (van den Heuvel et al., 2009). To conclude, these results support the hypothesis that localization-related epilepsy leads to cognitive impairments by inducing global changes in the brain network instead of a localized disruption only.

Apart from TLE, other types of epilepsy such as childhood absence epilepsy (CAE) and sleep-related hypermotor epilepsy (SHE) have recently been investigated by researchers (Wang et al., 2017; Evangelisti et al., 2018). CAE is a common generalized epilepsy syndrome with a presumed genetic cause, characterized by episodes of sudden, profound impairment of consciousness without loss of body tone, appearing in otherwise healthy school-aged children. Wang et al. (2017) compared centrality measures between CAE patients and healthy controls and hypothesized that hub nodes inside the DMN and thalamus in CAE patients were clearly damaged. In other work, Evangelisti et al. (2018) reported topological alterations mainly in basal ganglia and limbic system in SHE patients.

#### Alzheimer's Disease

The AD is a chronic and progressive neurodegenerative disorder that leads to deficits in memory and cognitive brain functions (Albert et al., 2011). The AD can be described as a disconnection syndrome because of the altered structural and functional connectivity architecture of the brain in those suffering from this disease (Pievani et al., 2011). Aging is naturally associated with some cognitive decline, but if this inefficiency is exacerbated in an individual's brain, one could experience mild cognitive impairment (MCI), which is an intermediate phase between age-related cognitive decline and dementia (Petersen, 2002). Statistical surveys report that ~15% of adults over 65 years old experience MCI (amnestic MCI or non-amnestic MCI) and that more than half of these cases convert to dementia in 5 years (Farlow, 2009). Early detection of the AD in subjects with MCI can prevent the progression of these impairments via disease-modifying treatments (Allison et al., 2014). Fortunately, the combination of graph theory and rs-fMRI has been able to act as a disease biomarker and reveal large-scale disconnection that is present before onset of AD symptoms (Wang et al., 2013; Brier et al., 2014; Dai and He, 2014; Botha and Jones, 2018).

By examining the brain network characteristics on functional connectivity, researchers concluded that individuals with AD exhibited degeneration of specific brain hubs, reduced clustering coefficients and path lengths very close to the values of random networks (Supekar et al., 2008; Sanz-Arigita et al., 2010; Dai et al., 2015; delEtoile and Adeli, 2017), similar to the results of researchers who worked on other imaging modalities (de Haan et al., 2009, 2012; Stam et al., 2009; Kim et al., 2015; Jalili, 2017). Also, other studies revealed that cognitive impairment in the AD was associated with a weakness in modular interconnectivity and hubs destruction (Brier et al., 2014) and significant alterations within the default network (Toussaint et al., 2014; Zhong et al., 2014). These findings were in parallel with a global decrease in long-distance functional connections especially between frontal and caudal brain regions (Sanz-Arigita et al., 2010). On the whole, the degeneration and randomization of the brain functional architecture in patients with AD indicates a great loss of global information integration. These results are highly associated with the anterior-posterior disconnection phenomenon and its role in the AD.

Moreover, authors combined graph theoretical approaches with advanced machine learning methods (here, support vector machines) to explore functional brain network alterations and classify individuals with AD using rs-fMRI (Khazaee et al., 2015, 2016; Hojjati et al., 2017). Further, by conducting statistical analysis on the brain networks of individuals with MCI who converted to AD (MCI converter) and those with stable MCI (MCI non-converter), they identified areas underlying this conversion (Hojjati et al., 2017). To sum up, these papers highlighted the efficiency of combining graph theory and machine learning for early detection of AD based on rs-fMRI connectivity analysis.

#### Multiple Sclerosis

MS is a chronic, degenerative, and heterogeneous autoimmune disease of the central nervous system, leading to physical, mental, or psychiatric problems (Marrie, 2017). Functional recovery in MS is achieved by repair of damage through remyelination and functional reorganization, which are the striking hallmarks of this disease (Filippi and Agosta, 2009). Most studies of functional connectivity based on graph theory in MS include analysis of rs-fMRI data (Gamboa et al., 2014). In one such study, Schoonheim et al. (2014) sorted the brain regions of interest based on their connectivity patterns using eigenvector centrality mapping (ECM) and reported MS-related differences for centrality in specific regions. As a result, decreased ECM values in sensorimotor and ventral stream areas were associated with clinical disability. In contrast, the thalamus and posterior cingulate demonstrated increased centrality as well as higher connectivity to regions with low centrality. To this end, the authors suggested a rerouting of thalamic communications to overcome the continuous inflammatory activity.

In two other studies, Shu et al. (2016) and Liu et al. (2017) compared the topological changes of functional connectome in individuals with clinically isolated syndrome (i.e., the earliest stage of MS) and MS patients. Their graph-based results indicated that disrupted network organization emerged in the earliest stage of MS, with a lesser degree relative to MS. Also, the extent of network alterations was correlated with cognitive impairment and physical disability only in MS patients. Importantly, Eijlers et al. (2017) attempted to demonstrate how abnormalities in functional network hierarchy are related to cognitive impairment in MS patients. Patients were classified into three categories: cognitively impaired, mildly cognitively impaired, and cognitively preserved. The centrality indices indicated that the occipital, sensorimotor, and hippocampal areas for all three patient groups became less central than healthy controls, while cognitively impaired patients displayed extensive centrality growth in areas making up the DMN compared to other groups. Their results can be interpreted as reflecting the hallmark alterations in functional networks of cognitively impaired patients with increased relative importance (centrality) of the DMN.

Taken together, major changes in topological parameters of the brain network have been observed in the sensorimotor, cingulate, and frontotemporal cortex, as well as in the thalamus (Schoonheim et al., 2014, 2015; Tewarie et al., 2015; Faivre et al., 2016; Rocca et al., 2016; Eijlers et al., 2017). The thalamus is often known as a relay organ between several cortical and subcortical regions, taking part in a large variety of neurological functions such as motor, sensory, integrative, and higher cortical functions (Minagar et al., 2013). Thus, thalamic degeneration may lead to cognitive dysfunction and physical disability in patients with MS, even in the early stages of the disease (Benedict et al., 2013).

#### Autism Spectrum Disorder

ASD is a complex neurodevelopmental disability characterized by difficulties in communication and behavior (Roux et al., 2012). The increasing prevalence of ASD over the last decade has underlined the need for medical assessment to identify the symptoms and signs of this disorder (Johnson and Myers, 2007). However, there are possible challenges in autism screening because of the uncertainty associated with the symptoms and neurobiological properties (Ecker et al., 2013; Mastrovito et al., 2018). These properties lead to great heterogeneity in the subjects and are the reason for the spectrum of the disease (Lenroot and Yeung, 2013; Jeste and Geschwind, 2014).

The contribution of rs-fMRI studies based on graph theory for autism exploration is considerable (Redcay et al., 2013; Rudie et al., 2013; Di Martino et al., 2014; Keown et al., 2017; van den Heuvel et al., 2017; Kazeminejad and Sotero, 2018). Authors in Rudie et al. (2013) and Keown et al. (2017) compared the brain topology in patients with ASD and healthy controls. They concluded that modularity, clustering coefficient, and local efficiency are relatively reduced in ASD (i.e., inefficiency of information transmission in a particular module) while global communication efficiency is increased (shorter average path lengths). As another example, Redcay et al. (2013) observed an increase in betweenness centrality and local connections by analyzing the prefrontal brain areas in adolescents with ASD. Moreover, the structure of the hub nodes was significantly changed in ASD (Itahashi et al., 2014; Balardin et al., 2015). Altogether, abnormalities in the functional architecture of the autistic brain were reported in both local and global metrics. Considering the huge discrepancies between subjects regarding local parameters (Finn et al., 2015), it was unclear whether such local parameters can be applied alone as a biomarker for ASD screening. To answer this question, Sadeghi et al. (2017) examined both local and global parameters extracted from rs-fMRI data and observed that distinctive features were only among the local parameters.

#### Attention-Deficit/Hyperactivity Disorder

ADHD affects about 3–5% of children globally (Nair et al., 2006). Wang et al. (2009) were the first to explore the spontaneous connectivity patterns of whole-brain functional network in patients with ADHD and healthy controls using graph analysis of rs-fMRI. They reported that the functional networks in both groups represented an economic small-world behavior. However, the brain networks of ADHD children exhibited more-regular configurations with higher local efficiency and a trend toward decreased global efficiency relative to healthy subjects, indicating a developmental delay of whole-brain functional networks in this pathology (Wang et al., 2009; Cao et al., 2013, 2014a, 2016; van den Heuvel et al., 2017). In addition, by testing nodal properties, Wang et al. (2009) claimed that areas such as medial prefrontal, temporal, and occipital cortices experienced regional loss of efficiency, while increased nodal efficiency was found in the inferior frontal gyrus.

Delayed maturation has further been reported in structural MRI studies (Hoogman et al., 2017), as well as in default network connectivity in youth with ADHD (Fair et al., 2010). Maturation rate differences between brain hemispheres may also characterize the ADHD brain, given significantly different interhemispheric asymmetry patterns recently observed in ADHD youths (Douglas et al., 2018). Analyzing rs-fMRI, Fair et al. (2010) scrutinized interregional connectivity patterns within DMN and noticed decreased anterior-posterior connectivity in children with ADHD compared to healthy controls. In another study, Fair et al. (2013) conducted a regional connectivity analysis using degree index on the functional networks in children with two different ADHD presentations, i.e., inattentive and combined. While both subtypes exhibited some overlapping (particularly in the sensorimotor network), the combined ADHD exhibited atypical patterns in midline DMN components and the inattentive ADHD showed atypical connectivity within the dorsolateral prefrontal cortex and cerebellum. Contrary to the findings of children with ADHD, Cocchi et al. (2012) did not find any significant changes in global characteristics of the whole-brain functional networks in adults with ADHD compared to healthy controls.

Apart from the region-wise studies, Tomasi and Volkow (2012) computed the voxel-wise Pearson's correlations across all pairs of brain voxels in ADHD children and healthy controls from the ADHD-200 database (Milham et al., 2012). Then, they classified the coefficients into long-range and short-range based on the anatomical distance, which was followed by constructing the corresponding functional connectivity density. As a result, they revealed that ADHD children had weaker interconnectivity (both long- and short-range) in the DMN, dorsal attention network, and cerebellum, and stronger short-range connectivity within reward network (ventral striatum and orbitofrontal cortex). Alterations in DMN have also been reported in studies applying non-negative matrix factorization (Anderson et al., 2014). In another study, Di Martino et al. (2013) observed similar centrality abnormalities within the precuneus in both ADHD and ASD groups, whereas ADHD patients exhibited particularly higher-degree centrality in the right striatum/pallidum. Finally, Colby et al. (2012) presented a machine learning approach using the combination of functional and structural graph-based features, as well as demographic information, to predict status of patients with ADHD from healthy children in the ADHD-200 database (Milham et al., 2012).

By interpreting the above findings, it can be concluded that the functional connectomes of ADHD children had a tendency toward regular configurations (Wang et al., 2009), while ADHD adults had no significant difference in terms of global architecture with healthy individuals (Cocchi et al., 2012). Also, disturbed nodal properties were identified in both children and adults, particularly in the attention, default-mode, sensorimotor, striatum, and cerebellum networks (Wang et al., 2009; Fair et al., 2010, 2013; Cocchi et al., 2012; Tomasi and Volkow, 2012; Di Martino et al., 2013).

## Challenges and Future Directions

In general, the consistency of results across similar experiments that employed a graph theoretical approach indicates that this perspective is promising for establishing a comprehensive and sustainable model in future fMRI studies. However, it is sometimes difficult to integrate all of the reported findings an of pathological brain networks because the results do not coincide with each other when the factors affecting the experiments are different. For instance, patient demographic factors (such as age, gender, educational level, etc.), disease-specific characteristics (such as duration, course, severity, disability level, etc.), sample size, and network construction greatly vary across the studies. As an example of network construction, ignoring the negative entries in the connectivity matrix is very likely to result in the loss of valuable information (Shu et al., 2016). To overcome these heterogeneities and increase the reliability of the findings, more consistent comparisons can be made across the studies. In addition, there are several image repositories for pairwise studies in the area of brain network connectivity that can be explored by various packages based on graph theory (Rubinov and Sporns, 2010; Hosseini et al., 2012; Kruschwitz et al., 2015; Wang et al., 2015a; Mijalkov et al., 2017; Waller et al., 2018).

Although the importance of computational approaches in fMRI analysis has been evident over the last decade, it has not always matched the richness of fMRI data (Cohen et al., 2017). Early methods mostly neglected the ability of predictive models to better understand the distributed and dynamic nature of neural representations. Recently, several theory-driven techniques have commenced to highlight the salient role of machine learning, algorithmic optimization, and parallel computing in fMRI analysis (Cohen et al., 2017). Hence, adoption of modern techniques, such as multivoxel pattern analysis (MVPA), convolutional neural network (CNN), generative models, and real-time analysis, then aligning them with graph theoretical concepts might open a new generation of experiments that could transform our understanding of complex properties in the human brain networks.

Another challenge in graph theory research is developing a consensus about which of the brain parcellation schemes is optimal for defining network nodes and constructing the brain network (Hayasaka and Laurienti, 2010). Different parcellation methods may lead to different topological properties in the human brain networks, and the results depend on the network resolution. However, for better insight, one can appraise the reproducibility of the primary findings by applying multiple parcellation schemes at different spatial scales, particularly those with high resolution (Liu et al., 2017). Moreover, node specification in developmental research is extremely important as it is possible for nodes to be dissimilar across a sample, which may distort the brain network. Therefore, a fundamental condition for ensuring the reliability of graph analysis in brain connectivity studies is the precise definition of network nodes (Stanley et al., 2013), which itself requires the adoption of an appropriate parcellation strategy (Power et al., 2010, 2011).

Although structural pathways are thought to underlie functional connectivity patterns (Honey et al., 2009), one cannot claim that there is a one-to-one correspondence between topological properties in functional and structural organizations (Park and Friston, 2013; Wang et al., 2015b; Mash et al., 2018). In some neurological diseases such as schizophrenia, small-world network abnormalities may even display opposite directions over functional and structural organizations. Concerning this matter, van den Heuvel et al. recognized evidence of reduced local efficiency and segregation (i.e., clustering and modularity) together with increased global efficiency in several functional studies of schizophrenia. However, their review of structural studies resulted in contradictory findings, such as increased segregation along with reduced integration and global efficiency (Van Den Heuvel and Fornito, 2014). Moreover, Shu et al. (2016) examined the structural and functional disruptions in the earliest stage of MS and MS patients by combined use of DTI and rs-fMRI. Their study exhibited structural changes in the earliest stage of MS, while functional patterns remained stable at that stage. Hence, structure-function relationship studies are needed to help elucidate such existing deviations for future work.

The primary features of the small-world organization, i.e., high local clustering yet short characteristic path length, contribute to the efficient flow of information within interconnected complex systems, a pivotal role that can reveal discrepancies between groups or across conditions. However, most techniques that evaluate small-world properties in real-world systems face significant constraints, such as misdiagnosis of some regular lattices as a small-world structure, lack of attention to weighted graphs, as well as neglecting the variations in network density and connection strengths. Fortunately, researchers have made notable efforts in the past decade to resolve these limitations in complex networks by proposing novel small-world metrics (Rubinov and Sporns, 2010; Telesford et al., 2011; Bolaños et al., 2013; Muldoon et al., 2016). Applying these newly introduced measures into future brain connectivity investigations can bring about widespread improvement in knowledge regarding small-world brain architecture.

The dynamics of brain function seem to result in numerous cognitive, emotional, and behavioral changes that occur during brain development. However, the majority of studies cannot interpret brain network dynamics because their design is typically cross-sectional and the calculated measures of the brain graph are only capable of displaying a snapshot of the disease over time (Fleischer et al., 2017; Avena-Koenigsberger et al., 2018). Therefore, the progression of neurodegenerative disorders may not be well-understood, and subsequently, treatment strategies exhibit poor performance. Madhyastha et al. (2017) reported that longitudinal fMRI studies with graph theory provide a suitable means for understanding the development of pathological conditions, as well as tracking temporal correlations between topological alterations in the brain network. They also noted that some development-related issues are still not answered by existing software, which should be further explored (Madhyastha et al., 2017). Additionally, longitudinal studies could be employed in the future for monitoring brain network topological changes using different therapeutic strategies across longer time durations (Mears and Pollard, 2016).

## Conclusion

In this paper, we first reported an in-depth overview of the computational methods that were proposed to discover functional and effective connectivity in the human brain network using fMRI. In discussing each method, we highlighted their strengths and potential drawbacks. Then, as the main focus of the current paper, comprehensive information on graph theoretical analysis of connectivity patterns in the complex brain network along with its applications in neuroscience was presented. The brain network topology is expected to be responsive to cognitive performance, behavioral variability, experimental task, and neurological disorders such as epilepsy, Alzheimer's disease, multiple sclerosis, autism, and attention-deficit/hyperactivity disorder. Graph theoretical metrics such as node degree, clustering coefficient, average path length, hubs, centrality, modularity, robustness, and assortativity can be utilized to detect the topological patterns of brain networks and reflect cognitive and behavioral performances (Sporns et al., 2004; van den Heuvel et al., 2008b). However, graph analysis in human neuroscience faces a number of issues that remain unaddressed, restricting its interpretation and application (De Vico Fallani et al., 2014). Some examples are heterogeneity of the results, sensitivity to parcellation strategy and node specification, statistical variability of brain graphs due to noise, lack of attention to the structure-function relationship, neglecting the variations in network density and connection strength, and dynamics of the brain network. Addressing any of these limitations in future studies will help advance our understanding of functional neural networks in the human brain.

## Author Contributions

FF conducted the literature search and prepared the initial draft of the paper. WK supervised all aspects of manuscript preparations, revisions, editing, and final intellectual content. FF and WK were involved in study conception and contributed to intellectual content. NL contributed to intellectual content and edited the final draft of the paper.

### Conflict of Interest Statement

The authors declare that the research was conducted in the absence of any commercial or financial relationships that could be construed as a potential conflict of interest.
